# Synthesis of vanadium pentoxide doped zinc oxide nanocomposites via laser ablation and their antibacterial activity and cell viability

**DOI:** 10.1038/s41598-026-49830-3

**Published:** 2026-05-04

**Authors:** A. A. Menazea

**Affiliations:** Spectroscopy Department, Physics Research Institute, National Research Centre, Dokki, Giza, 12622 Egypt

**Keywords:** V_2_O_5_@ZnO, Green synthesis, Laser ablation, Antibacterial, Biotechnology, Chemistry, Materials science, Microbiology, Nanoscience and technology

## Abstract

This paper introduce new rout preparation of Zinc oxide nanoparticles (ZnO NPs) via green laser ablation. V_2_O_5_@ZnO was synthesized via co-precipitation. The fabricated ZnO and V_2_O_5_@ZnO have been annealed at 900 °C. In vitro antibacterial experiments have been conducted for the prepared samples against negative and positive organisms. Additionally, this study discusses the impact of vanadium oxide insertion on the cytotoxicity of zinc oxide toward the human osteoblast cell line. The value of cell viability for ZnO was around 78.72 ± 3.54 and improved to 89.64 ± 2.65% for V_2_O_5_@ZnO nanocomposite approve the good biocompatibility of samples. The antibacterial activity of V_2_O_5_@ZnO nanocomposite that has been fabricated via co-precipitation method and annealed at 900 °C has been enhanced. The inhibation zone against *B. subtilis*, *S. aureus*, *P. aeruginosa*, and *E. coli* have been modified to 17.57 ± 0.67, 10.94 ± 0.35, 18.26 ± 0.91, 17.72 ± 0.98, respectively suggesting the application of the fabricated V_2_O_5_@ZnO in biological use such as wound healing.

## Introduction

Recent research has focused on green synthesis tools to produce nanoparticle solutions to avoid environmental problems caused by industrial wastes worsen over time^1^. Many researchers created environmentally friendly nanoparticles from laser ablation^2–4^. Nanocomposites are utilized in various biomedical and medicinal applications^5,6^. As a result, the material prepared using green routes doesn’t contain any remaining bioactive substances, which may result in high non-toxicity behavior^7,8^. Because of their intriguing properties in photocatalytic, gas sensing, dye-sensitised solar cells, photovoltaic applications, and other fields, ZnO and V_2_O_5_ are the most sought-after components in nanoscience and technology^9,10^.

Our skin is a vital organ in our bodies. It shields the body from outside invasion. Wound healing is the process by which the skin is repaired and remodeled after it has been damaged^11,12^. It is made up of four overlapping steps, including molecular and physiological processes. Hemostasis, inflammatory, proliferation, and remodeling are all interleaved processes^13^. Many cells and growth factors are involved in these steps. Wound dressing is a natural product that heals wounds.

Infections cause a delay in the healing process^14^. *Pseudomonas aeruginosa* and *Staphylococcus aureus* are two microorganisms that can cause severe skin inflammation^15^. Deep within the body, microorganisms colonize when they are absorbed through wounds. The healing process is impacted by this^16^. It prolongs the process of regeneration and healing. There will be an extension of the inflammatory phase of tissue repair. This will cause a delay in the angiogenesis process. For the wound to heal, these infections must be treated^17^. Catalytic activity^18,19^ and antibacterial activity^20,21^ against bacteria that cause wound infection are two properties of nanomaterials. The development of metal oxide nanoparticles with carefully regulated size and structure has resulted in a novel class of nanomaterials^22–25^. Antimicrobial nanomaterials have piqued the interest of scientists in various biological applications^26^. The antibacterial activities of nanomaterials are because of their unique nanoscale characterization^27^. When the shape and size of nanoparticles are changed at the nano level, their properties improve^28^. The size of these metal oxide nanoparticles has a significant impact on their antimicrobial properties^29^. Because they can penetrate bacterial cells with pores on their surface the size of micrometers, size is crucial. The pores on the bacterial cell surface are nanometres. Hydrogen peroxide is produced by metal oxide nanoparticles^30^. This promotes cell growth. Metal oxide nanoparticles have this property, which can be used to heal wounds^31^. Metal oxide nanoparticles produce reactive oxygen species, which encourage fibroblast development^32^.

Zinc Oxide (ZnO) is a vital metal oxide that is used in a variety of biomedical applications^33^. The communication between nanoparticles and cells is critical in their biological applications. The biological and medical behaviour of ZnO nanoparticles is proportional to their size^34,35^. The formation of electron–hole pairs is responsible for ZnO’s biological applications. It aids in the re-epithelialization of tissue regeneration. Numerous techniques for producing nanocomposite powder have been proposed, including co-precipitation, sol–gel precipitation, solid-state reaction, and solution combustion^36^. Among such methods, the sol–gel precipitating strategy is the most popular for synthesizing nano-crystalline materials due to its ability to infer distinguishable metastable structure at low reaction temperatures and superb chemical homogeneity, with the additional benefit of preparing high purity and well-crystallised powder of the nanocomposites^37^. Metallic implants containing bioactive materials have several advantages, including greater corrosion resistance of implant surfaces and increased osteoconductivity with surrounding tissues^38^. The implant’s corrosion behavior and tissue biocompatibility are determined by the protective metal oxide thin coating, which is stable. When compared to other oxides, zinc oxide (ZnO) has a variety of advantages, including low toxicity, strong corrosion resistance, biocompatibility, and significant antibacterial activity^39–41^.

Due to its benefits over conventional techniques, the Pulsed Laser Ablation in Liquids (PLAL) process is the most efficient physical method for nano production^42^. This method’s simplicity and the absence of a chemical reagent solution are its advantages^43^. The PLAL technique is notable for its methodological simplicity, flexible physical procedure, and lack of chemical reagents in solutions as compared to other traditional methods for creating metal colloids^44^. This type of plasma, known as laser-induced plasma, is created instantly when the laser beam ablates the solid target’s surface. The thermodynamic state of the laser-induced plasma is entirely different since the plasma is instantly contained by the liquid medium following its creation in the liquid environment.

Transition metal toxicity varies greatly but often involves disrupting essential metal balance (homeostasis) and causing oxidative stress via reactive oxygen species (ROS) production, leading to cellular damage, inflammation, and various acute/chronic health issues like organ damage, neurological problems, and even cancer, with common culprits including^45^. Certain metals, such as titanium dioxide (TiO_2_), are less hazardous than others, such as copper oxide (CuO) or vanadium pentoxide (V_2_O_5_). Toxicity is largely dependent on the particular metal, its form (such as nanoparticles), dose, exposure route, and duration. Because of the unique features and benefits of ZnO nanoparticles, a variety of goods containing these nanoparticles are being provided to the service of humanity, and the effort required to manufacture them is expanding every day^46,47^. The ZnO nanoparticles were made utilizing the laser ablation in this work. The green synthesis process produces ZnO nanoparticles that are ecologically friendly, produce minimal waste, and are extremely safe and non-toxic^48^.

Herin, we introduce synthesis V_2_O_5_@ZnO composite at a high annealing temperature 900 °C. Zinc oxide NPs were synthesizedvia green laser ablation route and V_2_O_5_ doped zinc oxide via co-precipitation. It have been characterised structurally and morphologically. The research also investigates the cytotoxicity of the films towards the human osteoblast cell line to evaluate their biocompatibility. Moreover, in-vitro cell availability and antibacterial activity have been performed.

## Experimental work

### Materials

The high-purity (99.999%) zinc plate was acquired from Sigma-Aldrich. Vanadium pentoxide nanoparticles (V_2_O_5_ NPs) were bought from Merck Co.

### Synthesis of zinc oxide nanoparticles via laser ablation in liquids

The PLAL method has been used to manufacture ZnO NPs. A tiny beaker containing 30 ml of ultra-pure water was used to submerge a high-purity zinc plate. The ablation process has been powered by a Nd:YAG nanosecond laser with a wavelength of 1064 nm, a power output of 3.6 W, a pulse repetition rate of 10 Hz, and a pulse duration of 7 ns. The laser ablation method has been thoroughly examined before, and the experimental work has been explained. A convex lens with a focal length of 10 cm focused the laser beam perpendicularly on the zinc plate. The synthesized zinc oxide nanoparticles solution has been dried in oven to get ZnO NPs in powder form.

### Synthesis V_2_O_5_@ZnO nano-composite

To prepare make V_2_O_5_@ZnO nano-composite V_2_O_5_@ZnO, 3 gm of V_2_O_5_ and previously made ZnO were combined in identical conditions via co-precipitation method. To achieve high crystallinity, the powder of the synthesized pure ZnO NPs and the prepared V_2_O_5_@ZnO nano-composite via co-precipitation method have been annealed at 900 °C for 2 h at a rate of 10 °C/min.

### Characterization techniques

PANalytical X-Pert PRO XRD has been utilized for structural analysis of the prepared pure ZnO via green laser ablation route and the prepared V_2_O_5_@ZnO via co-precipitation method and annealed at 900 °C using a Copper target and K radiation of wavelength (λ = 1.5404 Å). The tube was operated at 30 kV with a Bragg angle (2 theta) of 5 to 80. The (Perkin-Elmer 2000) spectrometer has been utilized to record FT-IR spectroscopy data in the 4000–400 cm^−1^ range. Scanning electron microscope model (QUANTA-FEG250, Netherlands) was used to investigate the surface morphology.

### Cell viability test

The human osteoblast cell line (HFB4) has been used to study the cell viability of the synthesized pure ZnO using green laser ablation route and the manufactured V_2_O_5_@ZnO via co-precipitation technique and annealed at 900 °C under DMEM culturing conditions. These cell lines were obtained from the VACSERA-Cell Culture Unit, Cairo, Egypt. The cells have been grown on the samples in 24-well plates and then incubated at 37 °C for three days. MTT (3-(4,5-dimethylthiazol-2-yl)-2,5-diphenyltetrazolium bromide) has been injected into each well, followed by an optical analyzer to evaluate cell viability. The measurement has been repeated three times for obtaining the standard deviation. The statistical analysis was conducted using MedCalc software version 15.0, developed in Mariakerke, Belgium. The calculation below was used to calculate the proportion of viable cells compared to the total number of cells^49^.

1 $${\mathrm{Viability}}\left( {{\% }} \right) = \frac{{\text{Mean optical density of test samples}}}{{\text{Mean optical density of the control }}} \times 100$$

### Antibacterial test

The antibacterial behavior of the synthesized pure ZnO via green laser ablation route and the prepared V_2_O_5_@ZnO via co-precipitation method and annealed at 900 °C have been tested against Gram-positive bacteria; (*B. subtilis* NRRL B-94and* S. aureus* NRRL B-313) and against Gram-negative bacteria; (*P. aeruginosa* NRC B-32 and* E. coli* NRC B-3703) using agar plate method. The control samples have been considered as Amikacin (30 µg Oxoid lot no. 2288626) and Ciprofloxacin (5 µg Oxoid lot no. 2280856). In Petri dishes containing agar media, 5 mg/ml of the liquid samples have been implanted and cultivated for 20 h at 37 °C. To obtain positive and negative deviations, the last approach was repeated three times. The algorithm below was used to calculate the antimicrobial activity index %^50^:

2 $${{\% }}\;{\mathrm{Activity}}\;{\mathrm{Index}} = \frac{{{\mathrm{Zone}}\;{\mathrm{of}}\;{\mathrm{inhibition}}\;{\mathrm{by}}\;{\mathrm{test}}\;{\mathrm{compound}}\left( {{\mathrm{diameter}}} \right)}}{{{\mathrm{Zone}}\;{\mathrm{of}}\;{\mathrm{inhibition}}\;{\mathrm{by}}\;{\mathrm{standard}}\left( {{\mathrm{diameter}}} \right)}} \times 100$$

## Results and discussions

### FTIR

**Fig. 1:**
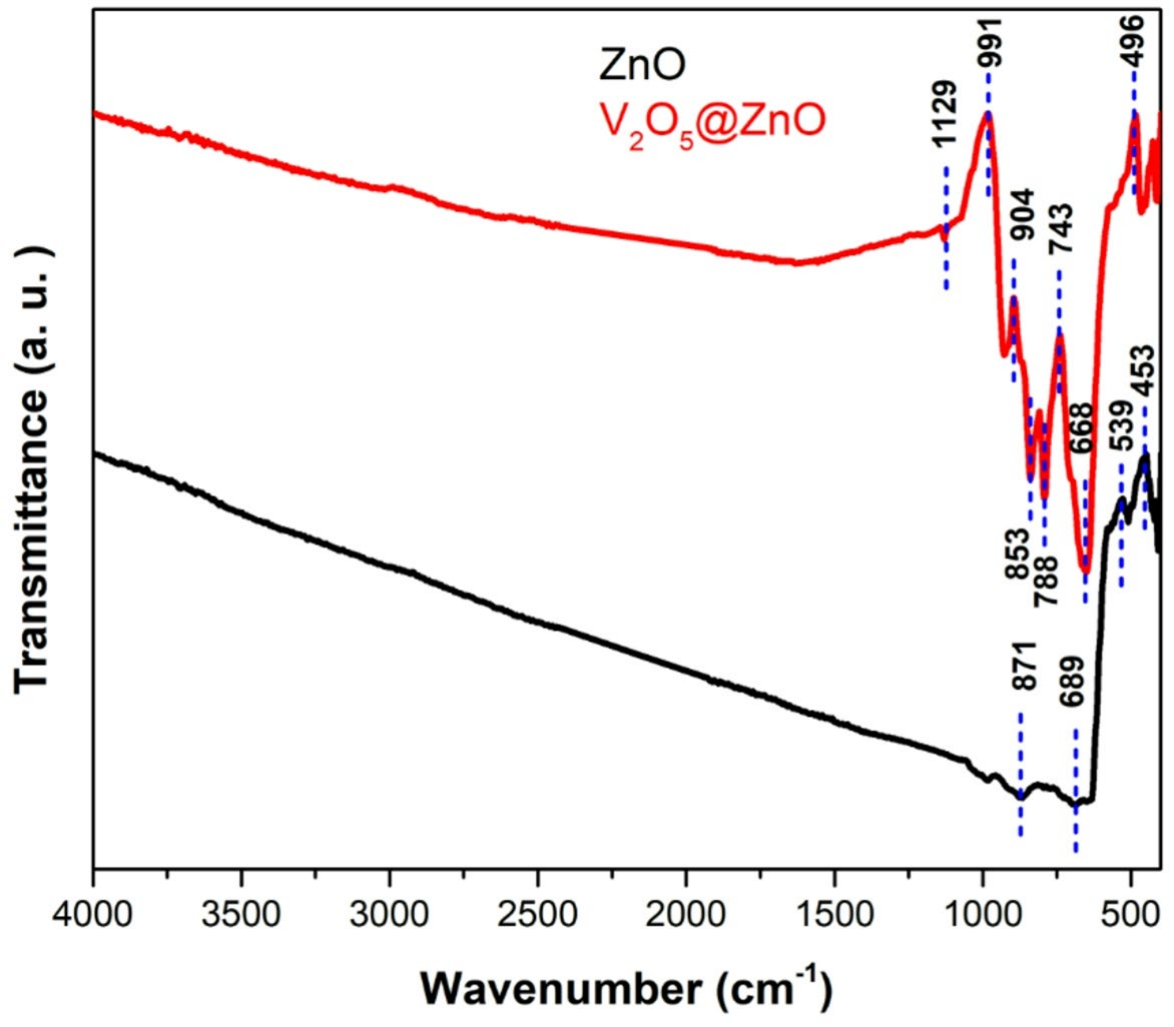
FT-IR spectra of the synthesized pure ZnO via green laser ablation route and the prepared V_2_O_5_@ZnO via co-precipitation method and annealed at 900 °C.

The composition of the synthesized samples has been performed and illustrated via FTIR technique in Fig. 1. The bands that illustrates at 871 cm^−1^, 453 cm^−1^ could be ascribed to vibrations of tetrahedral complexes and oxygen vacancy in ZnO, respectively^51^. The band is at 689 cm^−1^ because of the bending vibration^52^. The band at 539 cm^−1^ was due to (Zn–O) C–H vibrational mode^53^. For the sample V_2_O_5_@ZnO, the bands at 1129 and 991 cm^−1^ have been ascribed to (CO3)^2^ carbonate group^54^. The bands at 904, 853, 788, 743, and 668 cm^−1^ have been assigned to tetrahedral complex vibrations^55^. Bands centered on 496 cm^−1^ have been linked to oxygen vacancies in ZnO hexagonal symmetry^56^.

### XRD

**Fig. 2:**
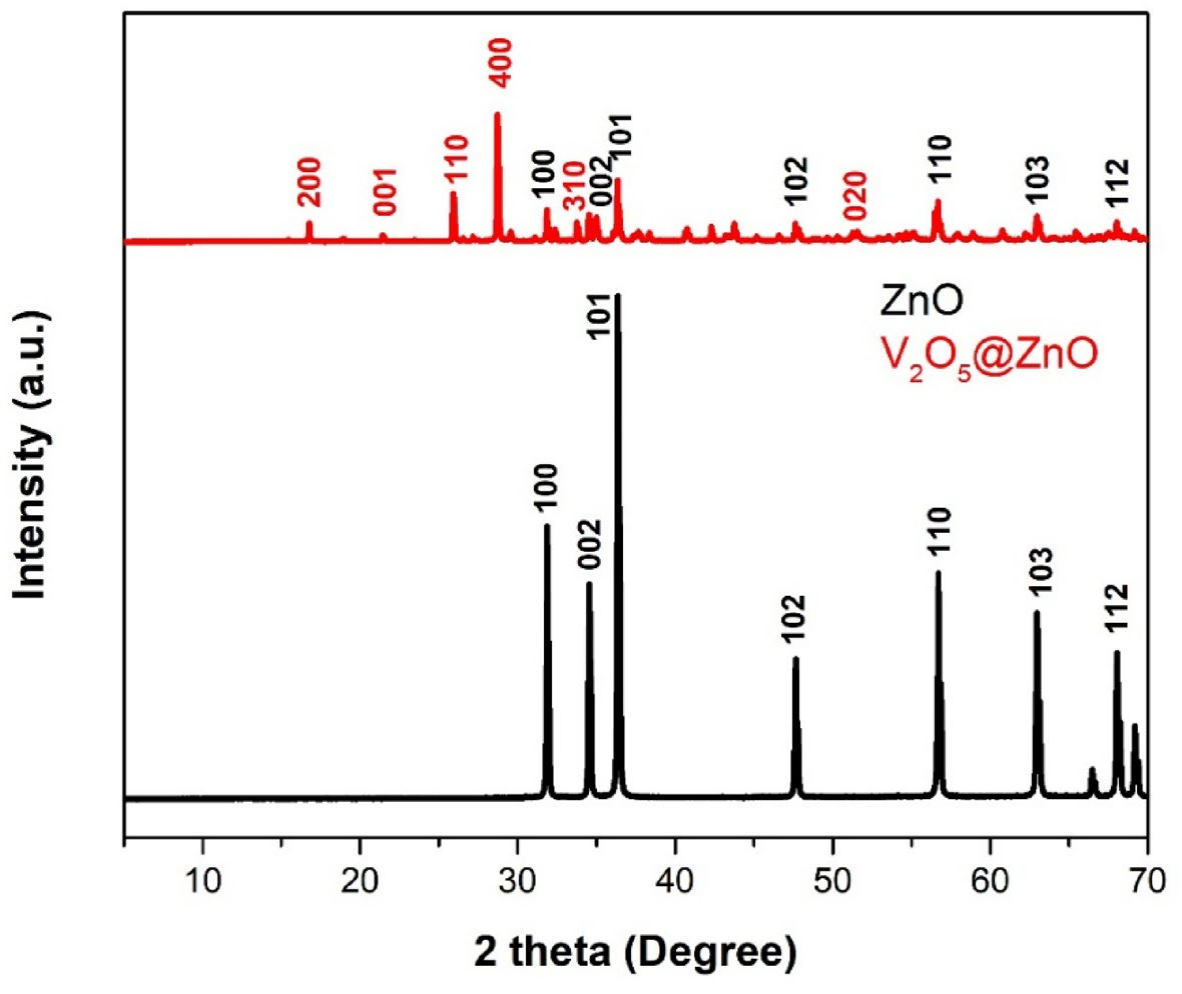
XRD pattern of the synthesized pure ZnO via green laser ablation route and the prepared V_2_O_5_@ZnO via co-precipitation method and annealed at 900 °C.

The structural characterization of the synthesized samples has been performed and illustrated via XRD technique. Figure 2 obtains XRD spectra of both ZnO and V_2_O_5_@ZnO samples. It shows diffraction peaks; the peak at 2θ = 31.76° corresponds to (100) plane, the peak at 34.47° corresponds to (002) plane, the peak at 36.41° corresponds to (101) plane, the peak at 47.64° corresponds to (102) plane, the peak at 56.74° corresponds to, (110) plane the peak at 62.93° corresponds to (103) plane and the peak at 67.97° corresponds to (112) plane. The structure of ZnO nanoparticles is characterized by these planes. As per the standard JCPDS file for ZnO (No. 89–1397), all the diffraction peaks matched well with a hexagonal structure of ZnO^57,58^. There were no additional impurity-related peaks visible, indicating that the high-purity ZnO nanoparticles were produced. In addition, the prepared V_2_O_5_@ZnO via co-precipitation method and annealed at 900 °C obtains the characteristic peak of ZnO NPs in addition the appearance of vanadium pentoxide NPs that was observed at 2θ values of 16.63°, 21.47°, 25.92°, 29.63°, 33.67° and 51.48°, which corresponding to the lattice planes (200), (001), (110), (400), (310), and (020) of orthorhombic V_2_O_5_ (JCPDS NO 41-1426)^59^.

The crystallite size of the synthesized samples has been recorded via debye- Scherrer’s equation as follows^59^:

3 $${\mathrm{D}} = {{{\mathrm{k}}\lambda } \mathord{\left/ {\vphantom {{{\mathrm{k}}\lambda } {\beta \cos \theta }}} \right. \kern-0pt} {\beta \cos \theta }}$$

where D is the crystallite size, $\uplambda$ is the wavelength, k is a constant value, $\upbeta$ is FWHM, and $\uptheta$ is Bragg’s angle. The crystallite size of zinc oxide is at about 32.74 nm, and for V_2_O_5_@ZnO the crystallite size enhanced to about 34.63 nm.

### Morphological investigations

**Fig. 3:**
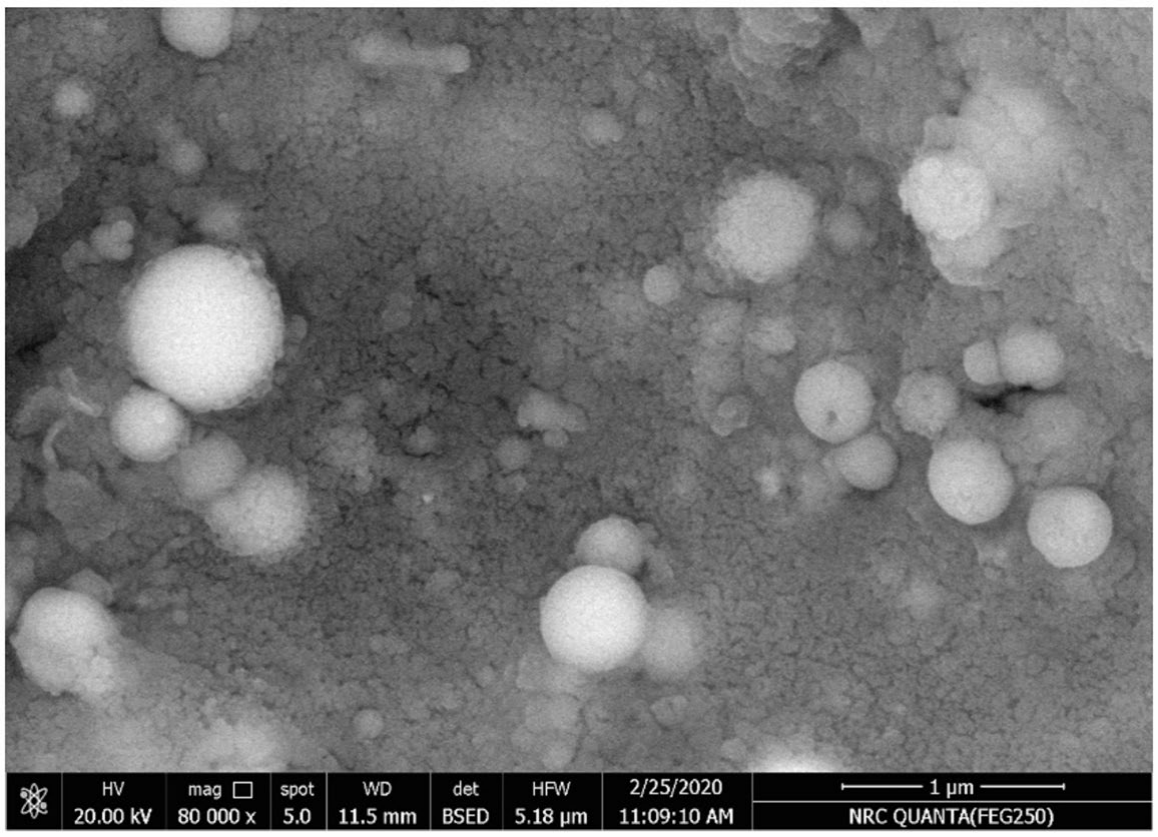
SEM scanning of the prepared V_2_O_5_@ZnO via co-precipitation method and annealed at 900 °C.

The morphological investigation of the synthesized samples was done with SEM analysis. Figure 3 shows the dispersion of small white spherical spots dispersed and distributed on the surface of the sample in addition to the appearance of semi-uniform big spherical which could be related to the agglomeration of vanadium pentoxide. Furthermore, the SEM image indicates that the development of ZnO could serves as the nano-composite, while V_2_O_5_ serve as fillers. As a result, the porosity could be reduced, thereby improving mechanical qualities.

### Cell viability

**Fig. 4:**
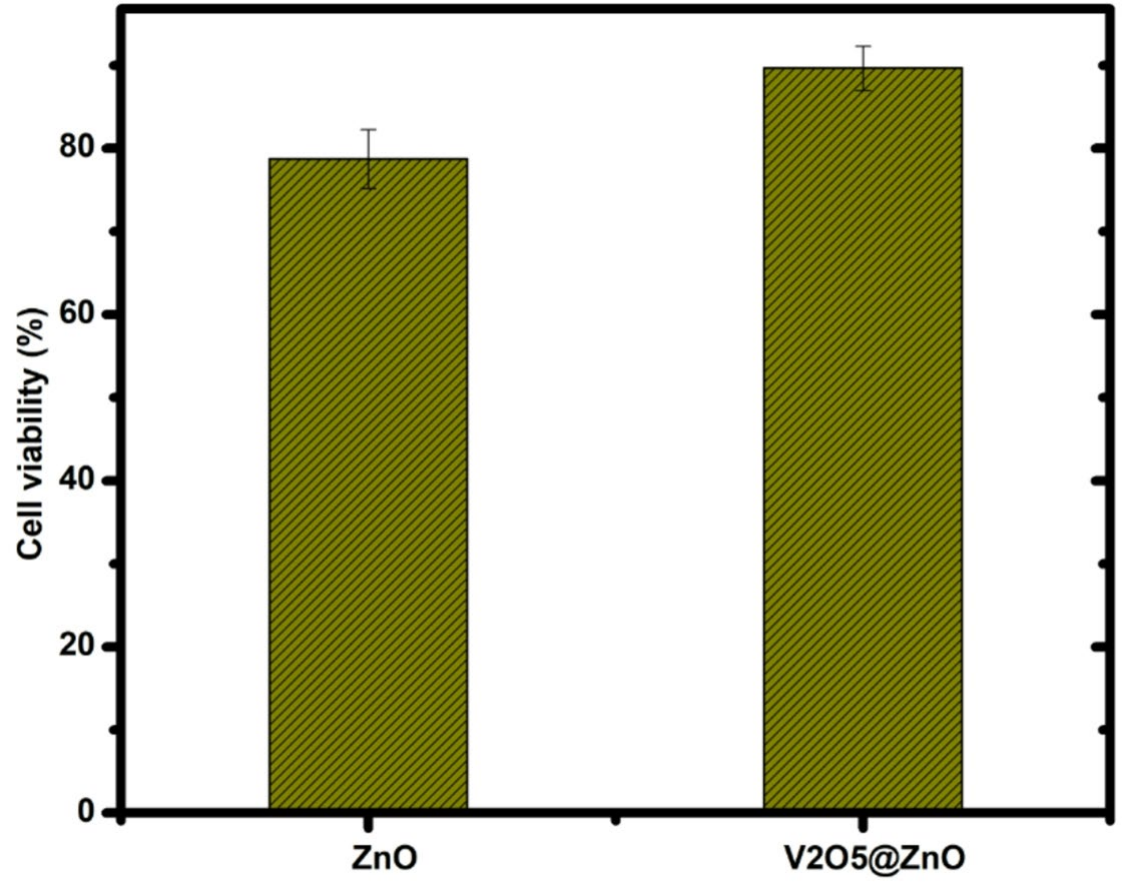
Cell viability of the synthesized pure ZnO via green laser ablation route and the prepared V_2_O_5_@ZnO via co-precipitation method and annealed at 900 °C.

This study attempts to investigate the effect adding vanadium pentoxide to the zinc oxide on its cytotoxicity towards a human lung cell line. The cell viability of the prepared samples has been obtained. Following two days of treatment, the MTT assay was utilized to assess the cells’ reaction to ZnO and V2O5@ZnO. In Fig. 4, the lowest cell viability ratio for pure ZnO was approximately 76.72%; however, it increased to 89.64% for V_2_O_5_@ZnO nanocomposite, indicating that both compositions are biocompatible. Additionally, this study explores the impact of vanadium pentoxide doping on the cytotoxicity of the zinc oxide towards a the human osteoblast cell line.

### Antibacterial studies

**Fig. 5:**
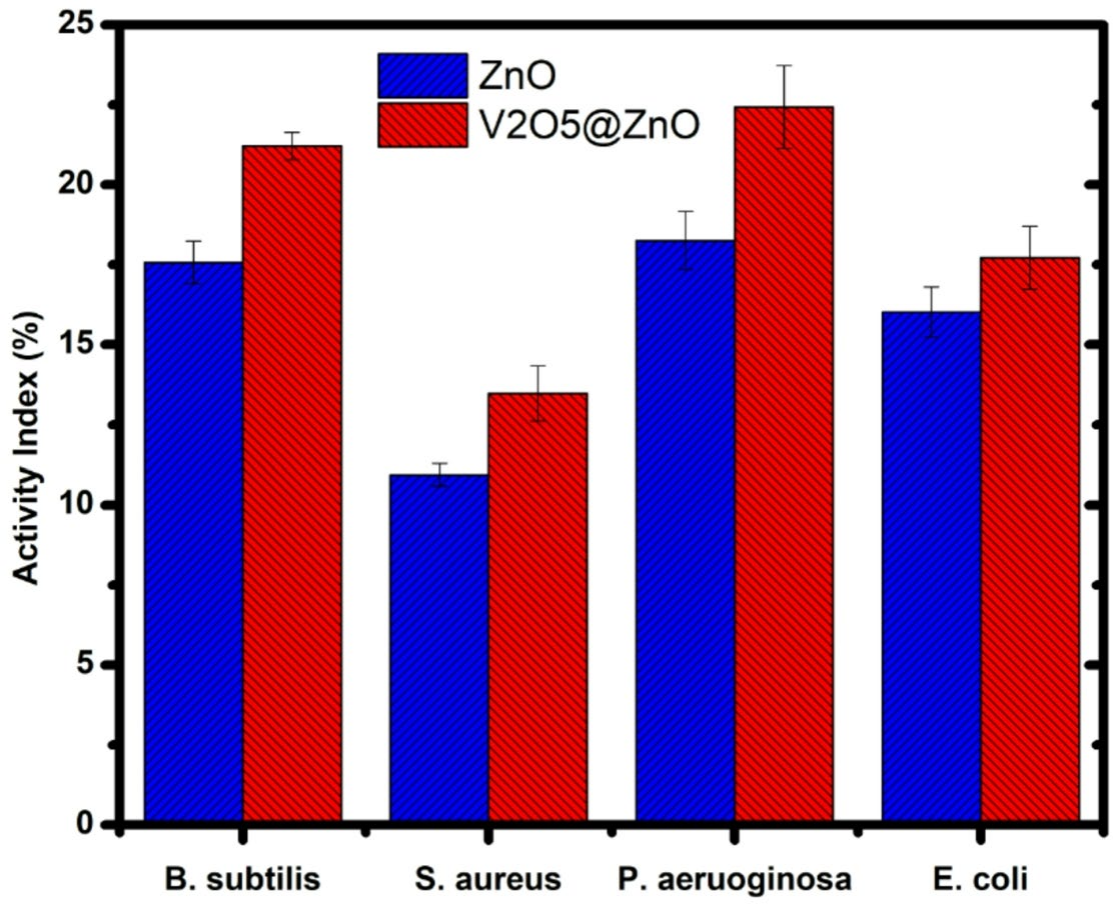
Antibacterial activity of the synthesized pure ZnO via green laser ablation route and the prepared V_2_O_5_@ZnO via co-precipitation method and annealed at 900 °C.

The antibacterial activity of the fabricated samples has been conducted against Gram-positive bacteria and Gram-negative bacteria using the agar plate method^60^. Table 1 and Fig. 5 illustrate how the antibacterial behaviour was ascertained by measuring the inhibition zone following a 24-h incubation period by Eq. 2. Good values were obtained in the inhibition zone of the synthesized pure ZnO via green laser ablation route and annealed at 900 °C; it was 21.21 ± 0.43 for B. subtilis, 13.47 ± 0.86 for S. aureus, 22.43 ± 1.3 for P. aeruginosa, and 16.02 ± 0.78 for E. coli. Additionally, by using a co-precipitation method and annealing the V2O5@ZnO at 900 °C, the inhibition zone was improved to 17.57 ± 0.67 for B. subtilis, 10.94 ± 0.35 for S. aureus, 18.26 ± 0.91 for P. aeruginosa, and 17.72 ± 0.98 for E. coli. Antibacterial inhibition refers to the formation of vanadium pentoxide nanoparticles from V_2_O_5_@ZnO that infiltrate into the agar layer and impede the formation of agar microbial colonies. We can conclude that all strains of B. subtilis, S. aureus, P. aeruginosa, and E. coli have an enhanced activity index when exposed to V2O5 nanoparticles doped with ZnO. After more investigation, the generated compositions’ enhanced antibacterial activity makes them a solid contender for use in microbial applications.

**Table 1:** The inhibation zone of ZnO NPs the fabricated via laser ablation route and V_2_O_5_@ZnO nanocomposite.

Type of bactaria	Zone of inhibition (mm)	
	ZnO	V_2_O_5_@ZnO
*B. subtilis*	17.57 ± 0.67	21.21 ± 0.43
*S. aureus*	10.94 ± 0.35	13.47 ± 0.86
*P. aeruginosa*	18.26 ± 0.91	22.43 ± 1.3
*E. coli*	16.02 ± 0.78	17.72 ± 0.98

## Conclusion

For biomedical applications, a metal oxide nanocomposite of vanadium pentoxide@zinc oxide (V_2_O_5_@ZnO) was synthesized at a high annealing temperature. Laser ablation route was used to make zinc oxide nanoparticles using a green technique. Co-precipitation was used to make V_2_O_5_@ZnO. ZnO and V_2_O_5_@ZnO were produced and annealed at 900°C. Antibacterial tests were performed in vitro on the produced samples against both negative and positive microorganisms. The cell viability ratio for pure ZnO was around 78.72 and improved to 89.64% for V_2_O_5_@ZnO nanocomposite, suggesting the good biocompatibility of both compositions The antibacterial activity of the fabricated V_2_O_5_@ZnO via co-precipitation method and annealed at 900 °C has been enhanced to; 17.57 ± 0.67 for *B. subtilis*, 10.94 ± 0.35 for *S. aureus*, 18.26 ± 0.91 for *P. aeruginosa*, and 17.72 ± 0.98 for *E. coli*. Greenly produced V_2_O_5_@ZnO composites free of toxic ingredients could be recommended for biological use.

## Data Availability

Data will be available on reasonable request from the corresponding author.
